# Inter-limb asymmetries in professional male basketball and volleyball players: bilateral vs. unilateral jump comparison

**DOI:** 10.1080/23335432.2025.2554589

**Published:** 2025-09-03

**Authors:** Dimitrije Cabarkapa, Damjana V. Cabarkapa, Andrew C. Fry

**Affiliations:** a Jayhawk Athletic Performance Laboratory – Wu Tsai Human Performance Alliance, Department of Health, Sport and Exercise Sciences, University of Kansas, Lawrence, KS, USA; b D2 Lab, Novi Sad, Serbia; c Faculty of Physical Education and Sports Management, Singidunum University, Belgrade, Serbia

**Keywords:** strength, injury, basketball, volleyball, biomechanics, ACL, rehabilitation, sports

## Abstract

The purpose of the present study was to examine differences in inter-limb asymmetries between countermovement vertical jump (CMJ) and single-leg jump (SLJ) performed on an innovative portable force plate system. Seventy professional athletes competing in top-tier international leagues (e.g. NBA, Super League) participated in this investigation. Following the completion of a standardized warm-up, athletes stepped on a uni-axial dual-force plate system and performed three CMJs and six SLJs without an arm swing in randomized order. Peak takeoff and landing forces were recorded for each limb, from which asymmetry percentages were derived. Wilcoxon signed-rank test was used to make statistical comparisons (*p* < 0.05). Significant differences were found in all asymmetry-related metrics, overall (CMJ vs. SLJ; takeoff: 7.2 vs. 1.0%; landing: 3.3 vs. 6.8%) and within each sport. Peak takeoff force asymmetry was considerably greater in CMJ than in SLJ, while peak landing force asymmetry displayed a reverse trend, with notably greater inter-limb asymmetries being detected within SLJ than CMJ. While both tests can offer useful information to sports practitioners, these results suggest that CMJ and SLJ should not be used interchangeably but rather in conjunction with one another to obtain a better insight into athletes’ performance capabilities and inter-limb asymmetry magnitudes.

## Introduction

Lower-limb asymmetries and their impact on athlete performance capabilities, injury risk, and return-to-play have been prominent research topics over the past decade (Bishop et al. 2019; Maloney 2019; Helme et al. 2021; Gao 2022). For example, a recently published study found an inverse relationship between asymmetries in peak velocity and power production during the Bulgarian split squat and change of direction performance (i.e. L-drill) within a cohort of collegiate American football players (Philipp et al. 2021). Similar observations were made by Bell et al. (2014) when studying 167 collegiate athletes participating in sports, such as volleyball, soccer, hockey, and golf, where an asymmetry in lower-limb power production greater than 10% resulted in a decreased jump height of approximately 9 cm. When examining inter-limb asymmetries and their association with injury risk within both athlete and non-athlete populations, a 15% threshold has often been used to classify individuals with a greater likelihood of injury occurrence than those that score below this threshold (Barber et al. 1990; Impellizzeri et al. 2007; Bishop et al. 2019; Cabarkapa et al. 2024a). Specifically, MacSweeney et al. (2024) found that kinetic asymmetries during the takeoff and landing phases of a double-leg countermovement vertical jump (CMJ) and jump height asymmetries in the single-leg vertical jump (SLJ) were directly associated with an increased risk of injury. In addition, in return-to-play scenarios, an arbitrary threshold of 10% has been used as an inter-limb asymmetry target for patient discharge and a marker of successful rehabilitation, especially for individuals who suffered anterior cruciate ligament tears (Rohman et al. 2015; Kyritsis et al. 2016; Bishop et al. 2019). However, this arbitrary threshold needs to be considered with caution as post-injury research designs often fail to establish if the inter-limb asymmetry was present before the injury occurrence, as well as its magnitude (Helme et al. 2021). In certain instances, athletes with quadriceps femoris strength asymmetry after anterior cruciate ligament reconstruction tend to display decreased knee-related function even after one-year post-operative procedures (Ithurburn et al. 2018).

While different lower-limb asymmetry testing protocols currently exist (e.g. back squat, isometric mid-thigh pull, isokinetic dynamometry), some of the most common methodologies implemented in an applied sports setting pertain to performing a CMJ or SLJ on uni-axial dual-force plate systems that offer a time-efficient neuromuscular performance analysis (Bishop et al. 2019; Cabarkapa et al. 2024a, 2024b, 2024c; Merrigan et al. 2024). The usage of these two testing protocols for asymmetry assessment purposes is further supported by the solid levels of reliability (e.g. strong intraclass correlation coefficient and small coefficient of variation) documented across several research reports (Bishop et al. 2019, 2021, 2022). However, it has been suggested that despite being similar tests at first sight, they should not be used interchangeably, as they offer different information to sports practitioners (Heil et al. 2020). For example, greater power outputs, jump height, and eccentric and concentric velocities have been observed in CMJ when compared to SLJ, as athletes can use two limbs instead of one to perform a specific task (Bishop et al. 2023). Conversely, due to the aforementioned differences, SLJ may be more suitable and sensitive in detecting asymmetries related to the strength and power-producing capabilities of the actual limb (Bishop et al. 2023). Thus, the results from the CMJ test should not be easily substituted with the results from the SLJ test.

Based on previously published research, peak force during takeoff and peak force during landing are some of the most commonly analyzed metrics pertaining to lower-limb asymmetry (Bishop et al. 2022; Gao 2022; Heil 2022). Also, the same two metrics are derived by software that accompanies many force plate systems (e.g. VALD Performance, Hawkin Dynamics), which are widely used by sports practitioners to assess athletes’ neuromuscular performance qualities acutely and over an extended period of time. One of the main reasons why these metrics tend to be within the main focus of sports practitioners is because the majority of non-contact injuries (e.g. knee and ankle) in team sports occur during dynamic body movements, such as jumping or landing maneuvers within passive joint tissue such as tendons or ligaments (Pedley et al. 2020; Heil 2022). So, the ability to adequately assess movement asymmetries is of critical importance to minimize the risk of injury and optimize on-court athletic performance.

Therefore, the purpose of the present study was to examine differences in lower-body asymmetry measures (i.e. peak takeoff and landing forces) assessed via CMJ and SLJ performed on an innovative portable force plate system within a cohort of professional basketball and volleyball athletes. Based on previously mentioned research reports, it is hypothesized that notable differences will be observed, which may provide sports practitioners with information that can be used for the improvement of assessment methodologies and ultimately training strategies.

## Methods

### Participants

A total of 70 professional athletes competing in top-tier international leagues (e.g. NBA, EuroLeague, SuperLeague) volunteered to participate in the present study, from which 35 were basketball (age = 25.7 ± 3.3 years; height = 200.8 ± 7.1 cm; body mass = 93.8 ± 16.5 kg) and 35 (age = 24.4 ± 3.9 years; height = 196.8 ± 5.2 cm; and body mass = 93.8 ± 16.5 kg) volleyball players. All athletes were active members of their teams and were cleared for participation in the team training activities by their respective sports medicine staff. No athlete reported any type of musculoskeletal injury or pain that could limit or impact jumping motion within a timeframe of 6 months prior to the testing. The testing protocol performed in the present study was approved by the University’s Institutional Review Board, and all athletes signed an informed consent document.

### Procedures

Before the start of the CMJ testing protocols, athletes completed a warm-up procedure, consisting of dynamic stretching exercises (e.g. A-skips, butt-kicks, high knees, side-to-side lunges, high-knee-pulls) and low-intensity sport-specific movements (e.g. lay-ups, partner passing) administered by their respective strength and conditioning coaches. Then, each athlete stepped on a uni-axial force plate system (ForceDecks Max, VALD Performance, Brisbane, Australia) and performed 3 non-consecutive CMJs and 6 SLJs (i.e. 3 with left leg and 3 with right) with no arm swing (i.e. hands on the hips during the entire movement). For the CMJ test, the athletes were instructed to stand on the force plates with feet shoulder-width apart, one foot on each plate, maintain a still posture for 1–2 s, and then perform a maximal vertical jump by dipping quickly and jumping straight up, keeping their hands on their hips throughout the movement and landing back on both feet before standing still again for 1–2 s. The same instructions were given during the SLJ tests, except athletes stood on one leg positioned in the center of the force plate and were instructed to keep the free leg from touching the ground or swinging excessively during the movement. The sampling frequency of the force plate system was 1000 Hz. To minimize a possible influence of fatigue, the jumps were performed in randomized order, and each jump was separated by a 20–30 s rest interval. Through the testing procedures, research assistants were present to provide strong verbal encouragement and ensure that the athletes followed the testing guidelines.

### Variables

Ground reaction force curves were derived via data analysis software (ForceDecks Max, VALD Performance, Brisbane, Australia) from which peak takeoff force (i.e. the highest force registered before takeoff within the concentric phase of the movement) and the peak landing force (i.e. highest force registered during the landing phase of the movement) were determined for both CMJ and SLJ. The selection of these performance metrics was based on previously published research reports (Bishop et al. 2022; Cabarkapa et al. 2024b). The takeoff and landing time points were determined as the values on the ground reaction force curve at which the force dropped below and rose above the 20 N threshold, respectively. The average value across the 3 jump trials for CMJ and SLJ was used for performance analysis. Then, the inter-limb asymmetry percentage was calculated (i.e. [L_leg_ − R_leg_/(L_leg _+ R_leg_/2)] × 100) for both types of jumping motions, overall and each sport separately.

### Statistical analysis

Descriptive statistics, mean and standard deviations ($\overset{ˉ}{x}$±SD) were calculated for CMJ and SLJ peak takeoff and landing forces, including 95% confidence intervals (CI). Shapiro−Wilk test and Q−Q plots were used to examine the assumption of normality. As inter-limb asymmetry percentages failed to meet the normal distribution, the Wilcoxon signed-rank test was used to make statistical comparisons between these two testing modalities. For those metrics, median and interquartile ranges were used for descriptive statistics purposes. The effect sizes were calculated as Z-statistic divided by the square root of the sample size (*r* = Z/√N), and they were interpreted as follows: <0.10 (trivial effect); 0.10–0.29 (small effect); 0.30–0.49 (moderate effect), and >0.5 (large effect). All statistical analysis procedures were completed in SPSS (Version 28.0; Chicago, IL, USA). The α level of *p* < 0.05 was used as a criterion for statistical significance.

## Results

Descriptive statistics, mean and standard deviations ($\overset{ˉ}{x}$±SD), for CMJ and SLJ peak takeoff and landing forces for each limb can be found in Table 1. Statistically significant differences were found in all asymmetry-related metrics (*p* < 0.05), overall and within each sport (i.e. basketball and volleyball). Peak takeoff force asymmetry was considerably greater in CMJ than in SLJ, with considerable effect size magnitudes (*r* ~ 0.800). On the other hand, the peak landing force asymmetry displayed a trend, with notably greater inter-limb asymmetries being detected in SLJ than in CMJ, with slightly smaller effect size magnitudes (*r* ~ 0.500). The descriptive statistics, median and interquartile ranges, and statistical significance for inter-limb asymmetry comparisons are presented in Table 2.

**Table 1.** Descriptive statistics and 95% confidence intervals [95% CI], for countermovement vertical jump (CMJ) and single-leg jump (SLJ) peak takeoff and landing forces.

Variable [unit]	CMJ	SLJ
*Basketball*		
Peak takeoff force – R [N]	1170.7 ± 240.9 [1088.0–1253.5]	1833.7 ± 355.4 [1711.7–1955.8]
Peak takeoff force – L [N]	1148.9 ± 233.9 [1068.5–1229.2]	1826.9 ± 339.2 [1710.4–1943.4]
Peak landing force – R [N]	2643.4 ± 913.0 [2329.7–2957.0]	3372.2 ± 855.0 [3078.5–3665.9]
Peak landing force – L [N]	2558.9 ± 771.1 [2294.0–2823.8]	3417.7 ± 897.8 [3109.3–3726.1]
*Volleyball*		
Peak takeoff force – R [N]	1007.7 ± 147.8 [956.9–1058.4]	1591.2 ± 227.3 [1513.1–1669.2]
Peak takeoff force – L [N]	1006.7 ± 152.9 [954.2–1059.3]	1610.5 ± 222.4 [1534.1–1686.9]
Peak landing force – R [N]	2227.6 ± 636.3 [2008.8–2446.2]	2972.0 ± 554.5 [2781.5–3162.5]
Peak landing force – L [N]	2144.0 ± 658.4 [1917.8–2370.3]	2991.1 ± 498.2 [2819.9–3162.2]
*Overall*		
Peak takeoff force – R [N]	1089.2 ± 214.7 [1038.–1140.4]	1712.5 ± 320.3 [1636.1–1788.8]
Peak takeoff force – L [N]	1077.8 ± 208.9 [1028.0–1127.6]	1718.7 ± 304.9 [1646.0–1791.4]
Peak landing force – R [N]	2435.5 ± 808.8 [2242.6–2628.4]	3172.1 ± 743.2 [2994.9–3349.3]
Peak landing force – L [N]	2351.5 ± 741.8 [2174.6–2326.5]	3204.4 ± 752.1 [3025.1–3383.7]

R – right leg; L – left leg.

**Table 2.** Descriptive statistics, median and interquartile range, and statistical significance for differences in asymmetry percentages between countermovement vertical jump (CMJ) and single-leg jump (SLJ) testing modalities.

Variable [unit]	CMJ	SLJ	*p*-value [ES]
*Basketball*			
Peak takeoff force asymmetry [%]	7.1 (8.2)	0.9 (1.4)*	< 0.001 [0.792]
Peak landing force asymmetry [%]	3.0 (5.5)	6.3 (12.0)*	0.004 [0.490]
*Volleyball*			
Peak takeoff force asymmetry [%]	7.2 (6.9)	1.1 (1.0)*	< 0.001 [0.833]
Peak landing force asymmetry [%]	2.4 (5.4)	7.3 (10.4)*	0.003 [0.504]
*Overall*			
Peak takeoff force asymmetry [%]	7.2 (7.8)	1.0 (1.3)*	< 0.001 [0.807]
Peak landing force asymmetry [%]	3.3 (5.5)	6.8 (11.2)*	< 0.001 [0.511]

ES – effect size; (*) – significantly different when compared to CMJ (*p* < 0.05).

## Discussion

The purpose of the present investigation was to examine differences in lower-body asymmetry measures assessed via CMJ and SLJ performed on an innovative portable force plate system. To the best of our knowledge, such a study is the first one to be conducted on a cohort of elite athletes, including 70 professional male basketball and volleyball players. The results reveal a presence of moderate to large statistically significant differences in peak takeoff and landing forces, overall and within each sport. Specifically, the peak takeoff force asymmetry was greater in CMJ than in SLJ, while the peak landing force asymmetry displayed a reverse trend, with the asymmetry percentage being greater in SLJ than in CMJ. Thus, based on these findings, we can determine that CMJ and SLJ tests should not be used interchangeably in an applied sport setting when attempting to assess lower-limb asymmetries.

Previously published research has implied the presence of notable differences in kinetic and kinematic parameters between CMJ and SLJ (Van Soest et al. 1985; Taylor et al. 2016; Bishop et al. 2021, 2023), which further supports the observations made in the present study. For example, when studying well-trained male volleyball players, Van Soest et al. (1985) found that mean net torques in the hip and ankle, as well as the net power output in the ankle, were considerably higher in SLJ than in CMJ, likely attributed to greater activation (i.e. electromyography analysis) in the vastus lateralis and gastrocnemius muscles. Taylor et al. (2016) found that double-leg landing tests, commonly used for the assessment of anterior cruciate ligament injury risk in a clinical setting, demonstrated different biomechanical characteristics when compared to the single-leg landing tasks. Specifically, double-leg landings often show greater hip and knee flexion angles than single-leg landings, which are more common in many sports and are associated with a higher risk of anterior cruciate ligament injury (Taylor et al. 2016). Moreover, a recently published study by Bishop et al. (2021) found poor levels of agreement between CMJ and SLJ tests in mean force and concentric and eccentric impulse. Overall, the aforementioned research reports emphasize the notion that the nature of these two tests is very different, as our results indicate as well. Therefore, the takeoff and landing asymmetry percentages in peak force production need to be interpreted in conjunction with each other, rather than being substituted for one another.

Another interesting observation pertaining to the results obtained in the present study is that, regardless of the sport that athletes are participating in (i.e. basketball, volleyball), the asymmetry in force production during takeoff was greater in CMJ than SLJ. This may be attributed to the compensatory strategies in jumping mechanics that athletes may use while performing a specific jumping task (Bishop et al. 2021). For example, if an athlete has been dealing with a certain lower-body injury (e.g. mild ankle sprain), they may alter their jumping technique to avoid placing too much pressure on an injured limb during CMJ. This can ultimately result in improper performance assessment, as bilateral jumping tasks allow athletes to mask these asymmetries and utilize compensatory movement strategies (Taylor et al. 2016: Bishop et al. 2021). In addition, the same assumption applies when assessing lower-limb asymmetries in peak force production during bilateral landing tasks. Specifically, Hua-Yeow et al. (2011) have found considerably different energy dispersion strategies between double-leg and single-leg landings in both sagittal and frontal planes of motion. These findings seem to agree with the results obtained in the present study, where significant asymmetries in peak force production during the landing phase of the movement were observed between CMJ and SLJ. Therefore, as previously indicated, while both testing modalities offer relevant information to sports practitioners, they should complement each other, rather than be used interchangeably.

Although both CMJ and SLJ demonstrated solid reliability levels (Bishop et al. 2019, 2021, 2022), the choice of test should be based on the sport in which the athlete is participating. Considering that both basketball and volleyball are team sports that require athletes to perform repetitive bilateral movements (Mihajlovic et al. 2023; Yin et al. 2023; Cabarkapa et al. 2024b, 2024c), the CMJ might be a test for the lower-limb asymmetry assessment. However, based on previously mentioned scientific literature (Van Soest et al. 1985; Taylor et al. 2016; Maloney 2019; Bishop et al. 2022) and the results obtained in the present study, sports practitioners working with this specific group of athletes should strive to complement the CMJ with the SLJ test to obtain a more comprehensive insight into athletes’ performance capabilities as well as a better assessment of the likelihood of injury occurrence.

While certain inter-limb asymmetries, particularly when pronounced or accompanied by underlying functional deficits, have been linked to an elevated risk of injury (Barber et al. 1990; Impellizzeri et al. 2007; Bishop et al. 2019; MacSweeney et al. 2024), it is important to recognize that bilateral asymmetries are prevalent among athletes across various sports (Afonso et al. 2022). Notably, there is currently no conclusive evidence indicating that the mere presence of asymmetry, especially within a moderate range, independently increases injury risk (Afonso et al. 2022). Some asymmetries may represent sport-specific adaptations or functional strategies developed over time in response to training or competition demands (e.g. baseball pitching, tennis swing). Therefore, asymmetries should be interpreted within the broader context of an athlete’s performance capacity, training background, and sport-specific demands, rather than being considered inherently problematic (Dominguez-Navarro et al. 2024).

Lastly, another important factor that needs to be considered when attempting to assess lower-body asymmetries in force production by using force plate systems is the impact of the learning effect (Cabarkapa et al. 2024b, 2024d). In certain instances, researchers have found fluctuations in athletes’ performance capabilities in various physical tests (e.g. Wingate anaerobic test, one-repetition maximum) in the absence of familiarization or a previous experience in performing the specific test (Ploutz-Snyder and Giamis 2001; Barfield et al. 2002). However, within a group of well-trained athletes, such as the ones examined in the present study, that constantly perform CMJ and SLJ as a part of their regular training sessions, this was found not to be an issue (Cabarkapa et al. 2024b, 2024e). Precisely, previous research has found stable performance measures in well-trained populations in force and power-producing capabilities across multiple testing sessions (e.g. eccentric mean force, concentric peak power, contraction time).

While offering a deeper insight into lower-limb asymmetry differences between CMJ and SLJ testing modalities, commonly performed in an applied sports setting, this study is not without limitations. The cohort of participants examined in this investigation was homogenous, as it consisted of highly trained male athletes participating in team sports, such as basketball and volleyball. Hence, future research is warranted to examine if these results are sex-specific and if they remain applicable to other individual and team sports (e.g. tennis, baseball, track and field), where the presence of unilateral sport-specific movements is more dominant. Also, further research should focus on examining how these asymmetry percentages change over the competitive season span or throughout the return-to-play rehabilitation process, as well as for populations with musculoskeletal injuries at the time of data collection, including electromyography data.

## Practical application

The results of this study reveal a presence of moderate to large statistically significant differences in peak takeoff and landing forces when using innovative dual-force plate systems for analyzing lower-limb asymmetries within a group of elite professional athletes. Specifically, the peak takeoff force asymmetry was found to be greater in CMJ than in SLJ, while the peak landing force asymmetry displayed a reverse trend, with the asymmetry percentage being greater in SLJ than in CMJ. While both tests can offer useful information to sports practitioners, these findings suggest that CMJ and SLJ should not be used interchangeably when attempting to assess lower-limb asymmetries. Although CMJ may be a preferred test in team sports, such as basketball and volleyball, sports practitioners should strive to complement CMJ with SLJ to obtain a better insight into athletes’ performance capabilities and the likelihood of injury occurrence.
